# Comparative efficacy and acceptability of methylphenidate and atomoxetine in treatment of attention deficit hyperactivity disorder in children and adolescents: a meta-analysis

**DOI:** 10.1186/1471-244X-11-176

**Published:** 2011-11-10

**Authors:** Raveen Hanwella, Madhri Senanayake, Varuni de Silva

**Affiliations:** 1 Department of Psychological Medicine, Faculty of Medicine, University of Colombo, Sri Lanka

## Abstract

**Background:**

Psychostimulants and non stimulants are effective in the treatment of ADHD. Efficacy of both methylphenidate and atomoxetine has been established in placebo controlled trials. Direct comparison of efficacy is now possible due to availability of results from several head-to-head trials of these two medications.

**Methods:**

All published, randomized, open label or double blind trials, comparing efficacy of methylphenidate with atomoxetine, in treatment of ADHD in children, diagnosed using DSM-IV™ criteria were included. The outcome studied was ADHDRS-IVParent:Inv score. The standardized mean difference (SMD) was used as a measure of effect size.

**Results:**

Nine randomized trials comparing methylphenidate and atomoxetine, with a total of 2762 participants were included. Meta-analysis did not find a significant difference in efficacy between methylphenidate and atomoxetine (SMD = 0.09, 95% CI -0.08-0.26) (Z = 1.06, p = 0.29). Synthesis of data from eight trials found no significant difference in response rates (RR = 0.93 95% CI 0.76-1.14, p = 0.49). Sub group analysis showed a significant standardized mean difference favouring OROS methylphenidate (SMD = 0.32, 95% CI 0.12-0.53 (Z = 3.05, p < 0.002). Immediate release methylphenidate was not superior to atomoxetine (SMD = -0.04, 95% CI -0.19-0.12) (Z = 0.46, p = 0.64). Excluding open label trials did not significantly alter the effect size (SMD = 0.08, 95% CI -0.04-0.21) (Z = 1.27, p = 0.20). All-cause discontinuation was used as a measure of acceptability. There was no significant difference in all cause discontinuation between atomoxetine and methylphenidate (RR 1.22, 95% CI 0.87-1.71). There was significant heterogeneity among the studies (p = 0.002, *I*^2 ^= 67%). Subgroup analysis demonstrated the heterogeneity to be due to the open label trials (p = 0.001, *I*^2 ^= 81%).

**Conclusions:**

In general atomoxetine and methylphenidate have comparable efficacy and equal acceptability in treatment of ADHD in children and adolescents. However OROS methylphenidate is more effective than atomoxetine and may be considered as first line treatment in treatment of ADHD in children and adolescents.

## Background

Pathophysiology of ADHD is multifactorial and its causal mechanisms have not precisely been established. However structural and functional imaging studies suggest that dysfunction of cingulate, frontal, and parietal cortical regions and imbalances in the dopaminergic and noradrenergic systems, contribute to the pathophysiology of ADHD [1,2].

It is characterized by inattention, hyperactivity and impulsivity. Estimates of worldwide prevalence of ADHD among school aged children vary from 2.4-19.8% [3-5]. Children with ADHD commonly exhibit disruptive behaviour in the classroom and underachieve academically. ADHD is associated with co-morbidities such as learning disorders, tics, anxiety, oppositional defiant disorder and conduct disorder [6]. In the long term, antisocial behavior, substance abuse, and a variety of problems related to conduct and learning can occur [7].

Both psychosocial interventions and pharmacological treatment are effective in reducing ADHD symptom frequency and severity [3]. Methylphenidate, a psychostimulant, is available in short and extended release forms. In addition methylphenidate transdermal system provides consistent delivery of medication over the course of the day, acting for approximately 12 hours. Other stimulants effective in treatment of ADHD are dexamphetamine and mixed amphetamine salts. Stimulant medication reduces over activity, impulsivity, inattention and improves problematic behaviours [8,9].

Several non-stimulant medications have been used in the treatment of ADHD. The non-stimulant atomoxetine was introduced in the United States in 2002. It is a selective norepinephrine reuptake inhibitor. Double blind studies have established its efficacy in treatment of ADHD [10]. Tricyclic antidepressants and bupropion are other non-stimulants which are effective in treatment of ADHD [11].

Efficacy of both methylphenidate and atomoxetine has been established in placebo controlled trials [12-15]. Direct comparison of efficacy is now possible due to availability of results from several head-to-head trials of these two medications. Individual studies may lack adequate power to demonstrate a difference in treatment effect between the two medications. Meta-analysis allows pooling of data from all head to head trials and the findings will help clinicians in selecting medications for treatment of ADHD.

## Methods

### Study eligibility

We included all published randomized, open label and double blind trials published in any language which compared efficacy of methylphenidate with atomoxetine in the treatment of ADHD diagnosed using the Diagnostic and Statistical Manual of Mental Disorders, Fourth Edition (DSM-IV™) criteria, in children and adolescents [16].

### Search strategy

A study protocol detailing sources of data, search strategy, outcome measures, study selection criteria and statistical analysis was developed.

Studies were identified by searches for the period January 1995-December 2010. We searched PubMed, the Cochrane Central Register of Controlled Trials and the Cochrane Database of Systematic reviews. We also looked at the references of selected full text articles. Search terms used were atomoxetine, tomoxetine, methylphenidate, attention deficit/hyperactivity disorder, ADHD, psychostimulants, stimulants, randomized controlled trial, trial, study.

### Study selection

**Figure 1:**
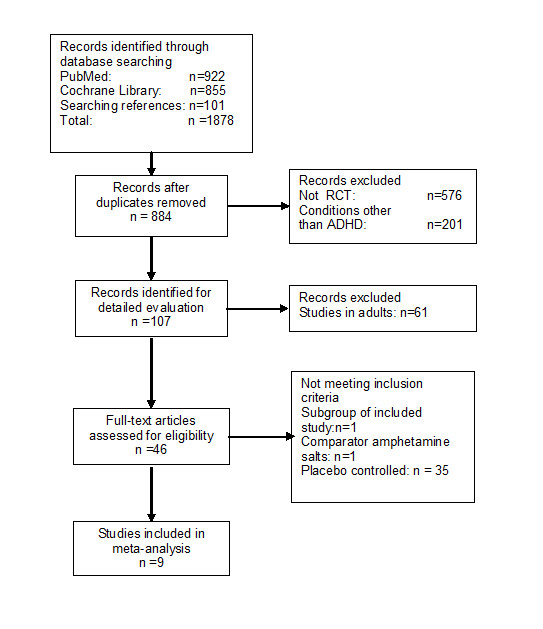
**Study flow summary**.

A trial flow summary is given in Figure 1. Titles and abstracts of selected studies were reviewed independently by two authors. Articles were selected for full text review if inclusion criteria were met. Disagreement about selection of articles was resolved by discussion between two authors and if agreement could not be reached, by the third author.

Methodological quality of the included studies was evaluated using the Detsky Quality Scale for Randomized Trials [17]. This scale gives a score ranging from 0-20 for positive trials and 0-21 for negative trials. The scale evaluates randomization, description of outcome measures, inclusion and exclusion criteria and description of therapy and statistics. Since all selected studies scored more than 12 on the Detsky quality scale, all were included in the meta-analysis.

### Data extraction

Data was extracted independently by two authors using a predesigned data extraction form. Title, year of publication, total number of participants, age of participants, design of study (parallel vs. crossover), blinding, name and dose of drug, number who dropped out and outcome measures were recorded. When data on standard deviations were missing, study authors were contacted to provide missing data [18] or it was calculated using the standard error of subgroups or confidence intervals [19-21]. Data was double entered by two authors.

### Statistical analysis

Outcomes studied were ADHDRS-IV Parent: Inv and Turgay DSM-IV-Based Child and Adolescent Behavior Disorders Screening and Rating Scale (T-DSM-IV-S) scores [22,23]. The standardized mean difference (SMD) was used as a measure of effect size. The ADHDRS-IV-Parent: Inv consists of 18 items corresponding to the 18 ADHD symptoms listed in the DSM-IV. Each item is rated on a scale from 0 = 'never/rarely' to 3 = 'very often.' The total score ranges from 0 to 54. The T-DSM-IV-S is based on the DSM-IV diagnostic criteria and assesses hyperactivity-impulsivity (9 items), inattention (9 items), opposition-defiance (8 items), and conduct disorder (15 items).

The relative risk of response was calculated. All cause discontinuation was used as a measure of acceptability. For these dichotomous outcomes, risk ratio (RR) was computed using the Mantel-Haenszel method. Data was analyzed using Review Man (RevMan) version 5.0 for Windows [24].

Meta-analysis was carried out using the random-effects model of DerSimonian and Laird [25]. The presence of heterogeneity between studies was tested using the Cochran's Q. The magnitude of heterogeneity was determined using the *I^2 ^*statistic.

There are reports that osmotically released methylphenidate is more effective than immediate release methylphenidate (IR MPH) [26]. Sub group analysis was conducted to examine whether treatment effect sizes varied between the formulations of methylphenidate. Because of the difference in methodological quality between double blind studies and open label trials, a sensitivity analysis was carried out excluding open label trials. One study used a cross-over design. As study results may be affected by a carry-over effect, we also conducted a sensitivity analysis excluding the cross-over trial.

## Results

### Study characteristics

Nine randomized trials comparing methylphenidate and atomoxetine in the treatment of ADHD in children and adolescents were identified (total participants 2762) [18-21,27-30]. The publication by Spencer et al. reported on two trials B4Z-MC-HFBD and B4Z-MC-HFBK [21]. Table 1 summarizes the characteristics of patients. Trial participants were aged 6-16 years. There were more male than female participants (77.6%) and majority was Caucasian (67%). Of the participants 47.4% had been previously treated with stimulants. Comorbid oppositional defiant disorder was diagnosed in 36%.

**Table 1:** Summary of patient characteristics

Characteristic	Atomoxetine	Methylphenidate	Total
**Age mean (SD)**			
**Gender n(%)**			
Male	1030 (79.8)	973(75.4)	2003(77.6)
Female	260(20.2)	317(25.6)	577 (22.4)
**Ethnic origin**			
Caucasian	871 (61.8)	946 (72.7)	1817(67.0)
Others	539 (38.2)	355 (27.3)	894(33.0)
**ADHD subtype**			
Hyperactive/impulsive	19 (2.3)	11 (2.0)	30 (2.2)
Inattentive	191 (23.3)	152 (28.3)	343(25.3)
combined	609 (74.4)	374 (69.7)	983 (72.5)
Prior stimulant use- yes	544 (55.0)	528 (41.4)	1072 (47.4)
Comorbid oppositional defiant disorder	273 (39.0)	134 (31.1)	407(36.0)

Table 2 summarizes the study characteristics. Five trials compared immediate release methylphenidate (IR MPH) with atomoxetine [18,21,27,29]. Three trials compared atomoxetine and osmotically released methylphenidate (OROS MPH) which is an extended release formulation [19,28,30]. One trial used both short acting methylphenidate and OROS MPH [20]. Eight trials employed a parallel design [19-21,27-30]. One trial employed a cross-over design [18]. In another trial which employed a cross-over design, only data from the first six weeks of treatment (parallel design) was included in the meta-analysis [28]. In the study which compared atomoxetine with standard treatment, only data for patients who received methylphenidate as their initial treatment was included in the analysis [20].

**Table 2:** Study characteristics

Study	Number of participants	Blinding	Design	Follow-up	Baseline severity ADHD-RS total score	Mean daily dose-atomoxetine and frequency	Mean daily dose-methylphenidate and frequency
Yildiz 2010	n = 25	Open label	Parallel group	12 weeks	Parents T-DSM-IV inattention scores atomoxetine = 16.72 MPH = 17.72	1.28 mg/kg/day Once a day	OROS-MPH = 1.07 mg/kg (IR = equivalent 0.89 mg/kg) Once a day
Newcorn 2008	n = 442	Double blind	Parallel group	6 weeks	Atomoxetine = 40.9(SD 8.8) MPH = 40.0 (SD 8.8)	1.45 mg/kg Twice a day	OROS-MPH = 1.16 mg/kg (IR equivalent = 0.966 mg/kg Once a day
Prasad 2007	n = 180	Open label	Parallel group	10 weeks	Atomoxetine = 45.5(SD 8.7) MPH not stated	1.5 mg/kg Once a daily 8 pts got twice daily	IR MPH = 0.8 mg/kg OROS-MPH = 1.03 mg/kg (IR equivalent = 0.858 mg/kg)
Wang 2007	n = 330	Double blind	Parallel group	8 weeks	Atomoxetine = 38.6 (SD 7.6) MPH = 37.4 (SD 7.6)	Final range 0.8 mg/kg-1.8 mg/kg Once a day	IR MPH = 17.8/mg/day Twice a day
Kemner 2005	n = 1323	Open label	Parallel group	3 weeks	Atomoxetine = 38.6 (SD8.1) MPH = 39.	1.08 mg/kg/Once a day	OROS-MPH 1.01 mg/kg/day (IR equivalent 0.841 mg/kg) Once a day
Sangal 2005	n = 85	Double blind	Cross over	7 weeks	Atomoxetine = 39.6 MPH not stated	1.56 mg/kg/day Twice a day	IR MPH = 1.12 mg/kg Three times a day
Kratochvil 2002	n = 228	Open label	Parallel group	10 weeks	Atomoxetine = 39.4 MPH = 37.6	0.48 mg/kg* or 1.4 mg/kg/kg** Twice daily	Final mean dose 0.85 mg/kg Three times a day
Spencer 2002 HFBD	n = 84	Double blind	Parallel group	9 weeks	Atomoxetine = 39.5	1.56 mg/kg Twice a day	IR MPH = 1.12 mg/kg Twice a day
Spencer 2002 HFBK	n = 79	Double blind	Parallel group	9 weeks	Atomoxetine = 39.5	1.56 mg/kg Twice a day	IR MPH = 1.12 mg/kg Twice a day

*atomoxetine slow metabolisers **atomoxetine rapid metabolisers

18 mg OROS MPH = 15 mg IR MPH

The final mean daily atomoxetine dose used in the trials ranged from 1.28 mg/kg-1.56 mg/kg. However one study used a smaller mean dose for patients who were identified as atomoxetine slow metabolizers [27]. Final mean daily dose for immediate release methylphenidate ranged from 0.8 mg/kg -1.12 mg/kg except for the study by Wang et al. which used a final dose range of 0.2-0.6 mg/kg. Mean dose of osmotically released methylphenidate when converted to immediate release dose equivalents ranged from 0.84 mg/kg 0.97 mg/kg. Atomoxetine was administered twice daily in five studies [18,21,27,28]. IR MPH was administered twice daily in two studies [21,29] while three studies administered thrice daily [18,21,27]. In one study the methylphenidate dosing schedule varied [20]. OROS MPH was administered once daily.

Duration of trials ranged from 3-10 weeks. Baseline severity of illness as measured by ADHD-RS scores ranged from 38.6-45.5 for atomoxetine and 37.4-40.0 for methylphenidate. One trial used Parents T-DSM-IV total to measure outcome and reported baseline severity of 44.2 (SD 7.5) for atomoxetine and 47.3 (SD 16.7) for MPH [30].

All studies except one excluded patients with tics. Patients with a history of bipolar disorder, psychosis, anxiety, seizures and current history or family history of Tourette syndrome were also used as exclusion criteria in several studies. Most of these are relative contraindications in clinical practice but are standard exclusion criteria in trials.

### Meta-analysis

**Figure 2:**
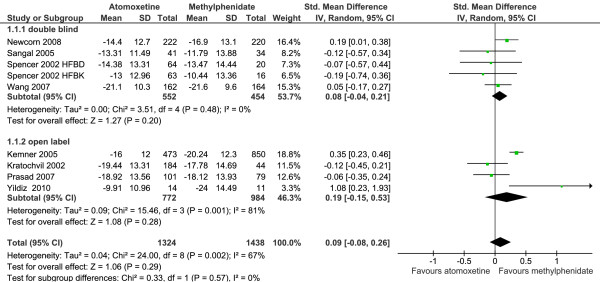
**Standardized mean difference in ADHDRS-IV scores for methylphenidate and atomoxetine**.

The results for the primary outcome are summarized in Figure 2. We used a random effects model in the meta-analysis because the subjects and interventions in the studies have differed in ways that would have impacted on the results. Meta-analysis did not find a significant difference in efficacy between methylphenidate and atomoxetine when standardized mean difference (SMD) was used as a measure of effect size 0.09 (95% CI -0.08-0.26) (Z = 1.06, p = 0.29).

### Response rate

Response rates were available for eight trials. Definition of response varied between trials. Five studies defined response as a ≥40% reduction from baseline to endpoint in the ADHDRS-IV-Parent:Inv [20,21,28,29]. One study defined response as a ≥25% reduction on the same scale [18]. For another study ≥50% reduction in scores was used as the definition of response [19]. One trial defined response as T-DSM-IV-S less than 40% of baseline scores [30]. There was no significant difference in response rates between the two medications (RR = 0.93 95% CI 0.76-1.14, p = 0.49).

### Sub group analysis

Sub group analysis showed a significant standardized mean difference favouring OROS methylphenidate 0.32 (95% CI 0.12-0.53 (Z = 3.05, p < 0.002). Immediate release methylphenidate was not superior to atomoxetine SMD -0.04 (95% CI -0.19-0.12) (Z = 0.46, p = 0.64).

### Sensitivity analysis

Excluding open label trials did not significantly alter the pooled standardized mean difference 0.08 (95% CI -0.04-0.21) (Z = 1.27, p = 0.20). Excluding the cross over trial too did not change the results significantly 0.11 (95% CI -0.07-0.29) (Z = 1.23 p = 0.22).

### Acceptability

Data from seven studies was available for assessment of all-cause discontinuation. There was no significant difference in all cause discontinuation between atomoxetine and methylphenidate (RR 1.22, 95% CI 0.87-1.71, p = 0.25).

### Heterogeneity

Heterogeneity is a measure of variation of effect size. *I*^2 ^is an estimation of the proportion of the observed variance reflecting real differences among studies. There was significant heterogeneity among the studies (p = 0.002, *I*^2 ^= 67%). Subgroup analysis found that the heterogeneity was due to the open label trials (p = 0.001, *I*^2 ^= 81%). There was no significant heterogeneity among double blind trials (p = 0.48 *I*^2 ^= 0%).

### Publication bias

Publication bias occurs if studies with small effect size or those showing no significant difference between the two medications are not published. However we were unable to carryout tests for funnel plot asymmetry due to the small number of studies.

## Discussion

This meta-analysis synthesized data from all trials comparing atomoxetine and methylphenidate in treatment of ADHD in children and adolescents. Placebo controlled trials have established that both methylphenidate and atomoxetine are effective in treatment of ADHD in children and adolescents [15,31,32]. This meta-analysis shows that in children and adolescents with ADHD, although the effect size favours methylphenidate the difference was not statistically significant. Subgroup analysis shows a significant standardized mean difference favouring OROS methylphenidate over atomoxetine. However immediate release methylphenidate was not superior to atomoxetine. There was no difference in acceptability as measured by all cause drop-out rate for methylphenidate and atomoxetine.

Two previous meta-analysis comparing atomoxetine and methylphenidate have included a smaller number of studies. Meta- analysis by Gibson et al. included five head to head trials comparing psychostimulants and atomoxetine [33,34]. The most recent meta-analysis excluded the largest studies by Kemner et al. as the study duration was only three weeks [34]. Our findings support those by Gibson et al. that OROS methylphenidate showed superior efficacy but there was no significant difference between atomoxetine and immediate release methylphenidate [33].

There is some evidence from randomized open label trials that OROS methylphenidate was superior to the immediate release form in treatment of ADHD [26,35]. However other studies have found no significant difference in efficacy between immediate release MPH three times a day and once daily OROS MPH [36-38].

Several methodological factors may have influenced the outcome of individual trials. The relatively lower efficacy of IR MPH may be explained by the dosing schedules. Only two studies administered an evening dose of IR MPH [16,25]. Symptom severity was assessed using the parent version of ADHDRS-IV and parents are likely to evaluate behaviours occurring outside school hours. The effect of IR MPH may wear off later in the day and this could account for the lower efficacy when IR MPH was administered only once or twice daily. Our meta-analysis found that the standardized mean difference in ADHD scores between atomoxetine and methylphenidate using parent ratings were relatively small and the difference may have been much larger if studies had used teacher ratings, because teacher ratings evaluate behavior during school hours. However only one study included in this meta-analysis used teacher ratings [30]. A meta-analysis by Cheng et al. showed that the effect size of atomoxetine using parent ratings was 0.34, about half of the effect size in teacher rating of 0.62 [39]. The meta-analysis reported a much smaller effect size for atomoxetine than that reported for MPH in studies using teacher ratings. Therefore the advantage of methylphenidate over atomoxetine appears larger in school than at home.

In contrast long acting OROS-methylphenidate which provides coverage in the late afternoon and evening may have an advantage over the immediate release form. The immediate release MPH equivalent does of OROS MPH used in the trials was 0.84 mg/kg-0.97 mg/kg. Therefore dose alone cannot account for the superiority of OROS methylphenidate.

Four trials used high doses of atomoxetine (> 1.4 mg/kg) administered twice daily [18,21,28]. The outcome in three of these trials favoured atomoxetine suggesting that higher doses of atomoxetine administered twice daily may be more effective.

Some design features would have been disadvantageous to atomoxetine. The trial with the largest number of participants was of short duration of three weeks [19]. This time period may not be adequate as optimal efficacy of atomoxetine requires 4-6 weeks of treatment [40]. Two trials excluded participants with previous poor response to methylphenidate, a design feature which would have favoured methylphenidate [27,28]. Although ADHD has high rates of comorbidity, subjects with tics, family history of Tourette syndrome and anxiety were excluded in most studies because methylphenidate use is contraindicated in these conditions. This may have exclude participants who are poor methylphenidate responders. Atomoxetine may not be as effective in patients with comorbid oppositional defiant disorder (ODD) [41]. Of all the trial participants 36% were diagnosed as having comorbid ODD.

Atomoxetine may be recommended for those with comorbid conditions where methylphenidate is contraindicated. Methylphenidate is contraindicated in individuals with structural cardiac abnormalities, arrhythmias, psychosis and suicidal ideation. However reports of suicidal ideation with atomoxetine and hepatic disorders have prompted warnings about the use of atomoxetine in these conditions [42]. In cardiovascular disease too, atomoxetine must be used with caution. There are concerns about the use of stimulants in children with comorbid seizure disorder and tics. There is evidence that in individuals with well controlled epilepsy, stimulants can be used safely and atomoxetine may also be associated with increased risk of seizures [43]. A recent review suggests that stimulants may not worsen tics in most with tic disorders [44].

The findings of this meta-analysis have to be interpreted cautiously because of several limitations. Interpretation of our findings is hampered by the substantial heterogeneity across studies. Sensitivity analysis showed the source of heterogeneity is the open label studies. Open label studies introduce bias as patient and investigator expectations can influence outcome. Because the number of studies within each subgroup is small there may not be adequate power to detect a difference between the two treatments. We did not include unpublished data from conference abstracts, dissertations and unpublished pharmaceutical company data, therefore publication bias is a distinct possibility. Tests for funnel plot asymmetry were not done as the Cochrane handbook for systematic reviews recommends that tests for funnel plot asymmetry should be used only when there are at least 10 studies included in the meta-analysis [45]. Since many of the trials excluded subpopulations with comorbid conditions the results of the meta-analysis cannot be generalized to these subpopulations. Seven studies were funded by Eli Lilly and Company manufacturers of atomoxetine and one study was sponsored by McNeil Consumer and Specialty Pharmaceuticals [19]. In addition several design features would have influenced the outcome of trials including non- inclusion of teacher rating scales which would have been a disadvantage for methylphenidate.

The findings of the meta-analysis have implications for clinical practice. The results from the meta-analysis show that in general atomoxetine and methylphenidate have comparable efficacy and equal acceptability in treatment of ADHD in children and adolescents. It also suggests that OROS methylphenidate is more effective and may be considered as first line treatment in treatment of ADHD in children and adolescents. Atomoxetine may be used in those with poor response to methylphenidate or where stimulant abuse is a cause for concern. Use of higher doses of atomoxetine administered twice daily may result in better outcome.

## Conclusions

In general atomoxetine and methylphenidate have comparable efficacy and equal acceptability in treatment of ADHD in children and adolescents. However OROS methylphenidate is more effective than atomoxetine and may be considered as first line treatment in treatment of ADHD in children and adolescents.

## Competing interests

RH and VdeS have received travel grants to attend academic meetings from Sun Pharma India. MS has no conflict of interest.

## Authors' contributions

All authors contributed to designing the study, data collection and analyzing the data. RH and VdeS drafted the manuscript. All authors read and approved the final manuscript.

## Pre-publication history

The pre-publication history for this paper can be accessed here:

http://www.biomedcentral.com/1471-244X/11/176/prepub

## References

[B1] Biederman J Attention-deficit/hyperactivity disorder: A selective overview Biol Psychiatry 2005 57 1215 1220 10.1016/j.biopsych.2004.10.020 15949990

[B2] Del Campo N Chamberlain SR Sahakian BJ Robbins TW The Roles of Dopamine and Noradrenaline in the Pathophysiology and Treatment of Attention-Deficit/Hyperactivity Disorder Biol Psychiatry 2011 69 12 e145 57 10.1016/j.biopsych.2011.02.036 21550021

[B3] American Association of Pediatrics Clinical practice guideline: diagnosis and evaluation of the child with attention-deficit/hyperactivity disorder. American Academy of Pediatrics Pediatrics 2000 105 5 1158 1170 10836893 10.1542/peds.105.5.1158

[B4] Centers for Disease Control and Prevention Mental health in the United States. Prevalence of diagnosis and medication treatment for attention-deficit/hyperactivity disorder--United States, 2003 MMWR Morb Mortal Wkly Rep 2005 54 34 842 847 16138075

[B5] Faraone SV Sergeant J Gillberg C Biederman J The worldwide prevalence of ADHD: is it an American condition? World Psychiatry 2003 2 2 104 113 16946911 PMC1525089

[B6] MTA Cooperative Group A 14-month randomized clinical trial of treatment strategies for attention-deficit/hyperactivity disorder. The MTA Cooperative Group. Multimodal Treatment Study of Children with ADHD Arch Gen Psychiatry 1999 56 12 1073 1086 10.1001/archpsyc.56.12.1073 10591283

[B7] Newcorn JH Advances in the Management of Attention-Deficit/Hyperactivity Disorder: An Evidence-Based Update Managed Care Consultant 2007 6 2

[B8] Kavale K The efficacy of stimulant drug treatment for hyperactivity: a meta-analysis J Learn Disabil 1982 15 5 280 289 10.1177/002221948201500508 6123539

[B9] Thurber S Walker CE Medication and hyperactivity: a meta-analysis J Gen Psychol 1983 108 1st Half 79 86 6131929 10.1080/00221309.1983.9711481

[B10] Hammerness P McCarthy K Mancuso E Gendron C Geller D Atomoxetine for the treatment of attention-deficit/hyperactivity disorder in children and adolescents: a review Neuropsychiatr Dis Treat 2009 5 215 226 19557116 10.2147/ndt.s3896 PMC2695220

[B11] Conners CK Casat CD Gualtieri CT Weller E Reader M Reiss A Weller RA Khayrallah M Ascher J Bupropion hydrochloride in attention deficit disorder with hyperactivity J Am Acad Child Adolesc Psychiatry 1996 35 10 1314 1321 10.1097/00004583-199610000-00018 8885585

[B12] Faraone SV Spencer T Aleardi M Pagano C Biederman J Meta-analysis of the efficacy of methylphenidate for treating adult attention-deficit/hyperactivity disorder J Clin Psychopharmacol 2004 24 1 24 29 10.1097/01.jcp.0000108984.11879.95 14709943

[B13] Faraone SV Biederman J Spencer TJ Aleardi M Comparing the efficacy of medications for ADHD using meta-analysis MedGenMed 2006 8 4 4 17415287 PMC1868385

[B14] Kratochvil CJ Milton DR Vaughan BS Greenhill LL Acute atomoxetine treatment of younger and older children with ADHD: a meta-analysis of tolerability and efficacy Child Adolesc Psychiatry Ment Health 2008 2 1 25 10.1186/1753-2000-2-25 18793405 PMC2556311

[B15] Schachter HM Pham B King J Langford S Moher D How efficacious and safe is short-acting methylphenidate for the treatment of attention-deficit disorder in children and adolescents? A meta-analysis CMAJ 2001 165 11 1475 1488 11762571 PMC81663

[B16] American Psychiatric Association Diagnostic and statistical manual of mental disorders 2000 4 American Psychiatric Association, Washington, DC

[B17] Detsky AS Naylor CD O'Rourke K McGeer AJ L'Abbe KA Incorporating variations in the quality of individual randomized trials into meta-analysis J Clin Epidemiol 1992 45 3 255 265 10.1016/0895-4356(92)90085-2 1569422

[B18] Sangal RB Owens J Allen AJ Sutton V Schuh K Kelsey D Effects of atomoxetine and methylphenidate on sleep in children with ADHD Sleep 2006 29 12 1573 1585 17252888 10.1093/sleep/29.12.1573

[B19] Kemner JE Starr HL Ciccone PE Hooper-Wood CG Crockett RS Outcomes of OROS methylphenidate compared with atomoxetine in children with ADHD: a multicenter, randomized prospective study Adv Ther 2005 22 5 498 512 10.1007/BF02849870 16418159

[B20] Prasad S Harpin V Poole L Zeitlin H Jamdar S Puvanendran K A multi-centre, randomised, open-label study of atomoxetine compared with standard current therapy in UK children and adolescents with attention-deficit/hyperactivity disorder (ADHD) Curr Med Res Opin 2007 23 2 379 394 10.1185/030079906X167309 17288692

[B21] Spencer T Heiligenstein JH Biederman J Faries DE Kratochvil CJ Conners CK Potter WZ Results from 2 proof-of-concept, placebo-controlled studies of atomoxetine in children with attention-deficit/hyperactivity disorder J Clin Psychiatry 2002 63 12 1140 1147 10.4088/JCP.v63n1209 12523874

[B22] DuPaul GJ Power TJ Anastopoulos AD Reid R ADHD rating scale-IV checklists, norms, and clinical interpretation 1998 New York: The Guilford Press

[B23] Turgay Disruptive Behavior Disorders Child and Adolescent Screening and Rating Scales for Children, Adolescents, Parents, and Teachers 1994 West Blomfield: Integrative Therapy Institute Publication

[B24] The Nordic Cochrane Centre Review Manager (RevMan) [ComputerProgram] Version 5.0 2008 Version 5.0 Copenhagen: The Cochrane Collaboration

[B25] DerSimonian R Laird N Meta-analysis in clinical trials Control Clin Trials 1986 7 3 177 188 10.1016/0197-2456(86)90046-2 3802833

[B26] Steele M Weiss M Swanson J Wang J Prinzo RS Binder CE A randomized, controlled effectiveness trial of OROS-methylphenidate compared to usual care with immediate-release methylphenidate in attention deficit-hyperactivity disorder Can J Clin Pharmacol 2006 13 1 e50 62 16456216

[B27] Kratochvil CJ Heiligenstein JH Dittmann R Spencer TJ Biederman J Wernicke J Newcorn JH Casat C Milton D Michelson D Atomoxetine and methylphenidate treatment in children with ADHD: a prospective, randomized, open-label trial J Am Acad Child Adolesc Psychiatry 2002 41 7 776 784 10.1097/00004583-200207000-00008 12108801

[B28] Newcorn JH Kratochvil CJ Allen AJ Casat CD Ruff DD Moore RJ Michelson D Atomoxetine and osmotically released methylphenidate for the treatment of attention deficit hyperactivity disorder: acute comparison and differential response Am J Psychiatry 2008 165 6 721 730 10.1176/appi.ajp.2007.05091676 18281409

[B29] Wang Y Zheng Y Du Y Song DH Shin YJ Cho SC Kim BN Ahn DH Marquez-Caraveo ME Gao H Atomoxetine versus methylphenidate in paediatric outpatients with attention deficit hyperactivity disorder: a randomized, double-blind comparison trial Aust N Z J Psychiatry 2007 41 3 222 230 10.1080/00048670601057767 17464703

[B30] Yildiz O Sismanlar SG Memik NC Karakaya I Agaoglu B Atomoxetine and Methylphenidate Treatment in Children with ADHD: The Efficacy, Tolerability and Effects on Executive Functions Child Psychiatry Hum Dev 2010 10.1007/s10578-010-0212-3 21165694

[B31] Cheng JY Chen RY Ko JS Ng EM Efficacy and safety of atomoxetine for attention-deficit/hyperactivity disorder in children and adolescents-meta-analysis and meta-regression analysis Psychopharmacology (Berl) 2007 194 2 197 209 10.1007/s00213-007-0840-x 17572882

[B32] Kratochvil CJ Wilens TE Greenhill LL Gao H Baker KD Feldman PD Gelowitz DL Effects of long-term atomoxetine treatment for young children with attention-deficit/hyperactivity disorder J Am Acad Child Adolesc Psychiatry 2006 45 8 919 927 10.1097/01.chi.0000222788.34229.68 16865034

[B33] Gibson AP Bettinger TL Patel NC Crismon ML Atomoxetine versus stimulants for treatment of attention deficit/hyperactivity disorder Ann Pharmacother 2006 40 6 1134 1142 10.1345/aph.1G582 16735655

[B34] Hazell PL Kohn MR Dickson R Walton RJ Granger RE van Wyk GW Core ADHD Symptom Improvement With Atomoxetine Versus Methylphenidate: A Direct Comparison Meta-Analysis J Atten Disord 2010 10.1177/1087054710379737 20837981

[B35] Remschmidt H Hoare P Ettrich C Rothenberger A Santosh P Schmidt M Spender Q Tamhne R Thompson M Tinline C Symptom control in children and adolescents with attention-deficit/hyperactivity disorder on switching from immediate-release MPH to OROS MPH Results of a 3-week open-label study Eur Child Adolesc Psychiatry 2005 14 6 297 304 10.1007/s00787-005-0467-6 16220213

[B36] Pelham WE Gnagy EM Burrows-Maclean L Williams A Fabiano GA Morrisey SM Chronis AM Forehand GL Nguyen CA Hoffman MT Once-a-day Concerta methylphenidate versus three-times-daily methylphenidate in laboratory and natural settings Pediatrics 2001 107 6 E105 10.1542/peds.107.6.e105 11389303

[B37] Wolraich ML Greenhill LL Pelham W Swanson J Wilens T Palumbo D Atkins M McBurnett K Bukstein O August G Randomized, controlled trial of oros methylphenidate once a day in children with attention-deficit/hyperactivity disorder Pediatrics 2001 108 4 883 892 10.1542/peds.108.4.883 11581440

[B38] Favreau A Deseille-Turlotte G Brault F Giraudeau B Krier C Barthez MA Castelnau P [Benefit of the extended-release methylphenidate formulations: a comparative study in childhood] Arch Pediatr 2006 13 5 442 448 10.1016/j.arcped.2006.02.004 16597499

[B39] Cheng JY Chen RY Ko JS Ng EM Efficacy and safety of atomoxetine for attention-deficit/hyperactivity disorder in children and adolescents-meta-analysis and meta-regression analysis Psychopharmacology (Berl) 2007 194 2 197 209 10.1007/s00213-007-0840-x 17572882

[B40] Michelson D Allen AJ Busner J Casat C Dunn D Kratochvil C Newcorn J Sallee FR Sangal RB Saylor K Once-daily atomoxetine treatment for children and adolescents with attention deficit hyperactivity disorder: a randomized, placebo-controlled study Am J Psychiatry 2002 159 11 1896 1901 10.1176/appi.ajp.159.11.1896 12411225

[B41] Bangs ME Hazell P Danckaerts M Hoare P Coghill DR Wehmeier PM Williams DW Moore RJ Levine L Atomoxetine for the treatment of attention-deficit/hyperactivity disorder and oppositional defiant disorder Pediatrics 2008 121 2 e314 320 10.1542/peds.2006-1880 18245404

[B42] Bangs ME Tauscher-Wisniewski S Polzer J Zhang S Acharya N Desaiah D Trzepacz PT Allen AJ Meta-analysis of suicide-related behavior events in patients treated with atomoxetine J Am Acad Child Adolesc Psychiatry 2008 47 2 209 18 10.1097/chi.0b013e31815d88b2 18176331

[B43] Graham J Coghill D Adverse Effects of Pharmacotherapies for Attention-Deficit Hyperactivity Disorder Epidemiology, Prevention and Management CNS Drugs 2008 22 3 213 237 10.2165/00023210-200822030-00003 18278977

[B44] Pringsheim T Steeves T Pharmacological treatment for Attention Deficit Hyperactivity Disorder (ADHD) in children with comorbid tic disorders Cochrane Database Syst Rev 2011 13 4 CD007990 10.1002/14651858.CD007990.pub2 21491404

[B45] Higgins JPT Green S Cochrane Collaboration Cochrane handbook for systematic reviews of interventions 2008 Chichester, England; Hoboken, NJ: Wiley-Blackwell

